# The Role of Estrogen Receptors and Their Signaling across Psychiatric Disorders

**DOI:** 10.3390/ijms22010373

**Published:** 2020-12-31

**Authors:** Wu Jeong Hwang, Tae Young Lee, Nahrie Suk Kim, Jun Soo Kwon

**Affiliations:** 1 Department of Brain and Cognitive Sciences, College of Natural Sciences, Seoul National University, Seoul 08826, Korea; hwj7942@gmail.com (W.J.H.); kwonjs@snu.ac.kr (J.S.K.); 2 Department of Psychiatry, Pusan National University Yangsan Hospital, Yangsan 50612, Korea; nahriekim@gmail.com; 3 Research Institute for Convergence of Biomedical Science and Technology, Pusan National University Yangsan Hospital, Yangsan 50612, Korea; 4 Department of Psychiatry, Seoul National University College of Medicine, Seoul 03080, Korea

**Keywords:** estrogen, estrogen receptors, schizophrenia, bipolar disorder, major depression disorder, autism, attention-deficit/hyperactivity disorder, raloxifene, hypothalamic-pituitary-gonadal axis

## Abstract

Increasing evidence suggests estrogen and estrogen signaling pathway disturbances across psychiatric disorders. Estrogens are not only crucial in sexual maturation and reproduction but are also highly involved in a wide range of brain functions, such as cognition, memory, neurodevelopment, and neuroplasticity. To add more, the recent findings of its neuroprotective and anti-inflammatory effects have grown interested in investigating its potential therapeutic use to psychiatric disorders. In this review, we analyze the emerging literature on estrogen receptors and psychiatric disorders in cellular, preclinical, and clinical studies. Specifically, we discuss the contribution of estrogen receptor and estrogen signaling to cognition and neuroprotection via mediating multiple neural systems, such as dopaminergic, serotonergic, and glutamatergic systems. Then, we assess their disruptions and their potential implications for pathophysiologies in psychiatric disorders. Further, in this review, current treatment strategies involving estrogen and estrogen signaling are evaluated to suggest a future direction in identifying novel treatment strategies in psychiatric disorders.

## 1. Introduction

Globally, one in seven people (equivalent to 11–18% of the population) suffers from mental or substance use disorders [1]. Despite many efforts, the prevalence of mental disorders remains high, and interestingly, there exist gender disparities. Women have a higher prevalence than men [1]. Indeed, multiple psychiatric disorders display sex differences in their symptoms, age of onset, and prevalence. In general, males are more susceptible to neurodevelopmental disorders, including schizophrenia, autism spectrum disorder (ASD), and attention-deficit/hyperactivity disorder (ADHD), whereas females are more susceptible to depressive, anxiety, and eating disorders. Multiple factors, such as social and environmental factors, via various pathways and circuits in the brain, play a role in creating these sex differences. However, accumulated evidence suggests biological factors as one of the strongest candidates underlying this phenomenon and a closer examination of sex hormones—in particular, estrogen.

Estrogens have traditionally been known to have their effects on reproductive behaviors, such as sexual receptivity and maternal behaviors [2]. However, over the past twenty years of extensive research, both in animals and humans, it is now known that estrogens, via their signaling mechanisms and interactions with multiple neurotransmitter systems in our brain, including dopamine, serotonin, and glutamate, have heavy involvement in cognition and mood [3,4,5]. Recent investigations have revealed pronounced interactions of estrogens with the dopaminergic system, a highly implicated system in the pathophysiology of multiple psychiatric and neurodegenerative disorders, and that they modulate executive functions, such as working memory and reward processing [6,7,8]. Further, the roles of estrogen receptors and estrogen signaling have been highlighted, with studies reporting their neuroprotective effects on the brain by promoting neurotrophins synthesis and protecting the brain from inflammation and stress [9,10,11]. To add more, investigations revealed, in animal models of psychiatric disorders and in patients, that estrogen and estrogen signaling are disturbed and that they are associated with not only the cognitive deficits but, also, the manifestations of the symptoms, which could also be reversed with estrogen administration or treatments targeting estrogen-signaling pathways [11,12,13]. Thus, together with much evidence on estrogen signaling disruptions in psychiatric disorders, recently, their effects have been taken under examination in multiple clinical trials for the critical assessment and evaluation of their efficacy as a new treatment for psychiatric patients [14,15,16,17,18]. Altogether, accumulating evidence suggests that estrogen and estrogen signaling may be highly implicated in the pathophysiology of psychiatric disorders, warranting a comprehensive and integrated understanding of estrogen and estrogen signaling across multiple levels of the brain system architecture from cellular and molecular to systemic to elucidate the mechanisms involved in its therapeutic effects in psychiatric disorders.

In this review, we first describe estrogen receptor signaling by providing summarized information of the literature on estrogen receptor signaling; distributions of estrogen receptors in the brain; their mechanisms of actions on major neurotransmitters of our brain, including dopaminergic, serotonergic, and glutamatergic; and their cognitive and neuroprotective effects. Next, we critically assess the recent progress of our understanding of the role of estrogen receptor signaling and its therapeutic effects in psychiatric disorders, including schizophrenia, bipolar disorder, major depressive disorder (MDD), ASD, ADHD, general anxiety disorder (GAD), post-traumatic stress disorder (PTSD), eating disorders, and substance use disorder, with an aim to provide and highlight the importance of estrogen singling in major psychiatric disorders, thereby possibly providing guidance as to finding new therapeutic targets.

## 2. Estrogen Receptor Signaling

### 2.1. Estrogen

The estrogen family is a steroid hormone and consists of one benzene ring, a phenolic hydroxyl group, and a ketone group, and, depending on the number of hydroxyl groups, the estrogens are named estrone (zero groups, E1), estradiol (one group, E2), estriol (two groups, E3), and estetrol (three groups, E4). While females produce estrogens all during their lives, however, for the predominance during the reproductive years and high relevance to physiology, the word estrogen in the literature commonly refers to E2 (or 17β-estradiol). Estrogens have been traditionally reported to have physiological functions involved in the development of breast tissue and sexual organs, regulations of the menstrual cycle and reproduction, and maintenance of our bone density. However, recent reports suggest its cognitive [3,19] and neuroprotective effects [10] and anti-inflammatory roles [20]. Estrogens are also present in males at low levels [21], and in men, they are involved in reproduction, such as spermatogenesis, erectile function, and libido [22]. Estrogens are produced primarily in ovaries from testosterone but can also be produced in the liver, adipose tissue, heart, and, most importantly, the brain [23]. In the brain, there exists regional specifics in estrogen production, suggesting their selective involvement of the brain functions. Reports show that estrogens are produced in the hippocampus, cerebellum, hypothalamus, amygdala, and cortex [24] by neurons and astrocytes [25].

### 2.2. Estrogen Receptors and Their Signaling Mechanisms

Estrogens exert their effects via estrogen receptors. There currently are three known classes of receptors, estrogen receptor alpha (ERα), estrogen receptor beta (ERβ), and G protein-coupled receptor 30 (GPER). With GPER being relatively recently discovered [26], ERα and ERβ are the most widely studied in the literature. ERα and ERβ are composed of various functional domains and have several structural regions in common, the amino-terminal domain (NTD) and estrogen response elements (ERE). Since estrogens are steroid hormones, they can exert their direct effects by entering the plasma membrane and taking estrogen receptor complexes to the cell nucleus and interacting and binding directly onto the ERE of intracellular ERα and ERβ. Otherwise, they can indirectly exert their effects by activating intracellular signaling cascades via interacting with estrogen receptors. Thus, estrogen signaling can be divided into genomic (direct binding onto ERE) and nongenomic (activation of an intracellular signaling cascade). Recent reports suggest that 35% of genes that are regulated by estrogen receptors lack EREs, in which only nongenomic estrogen signaling can be conducted [27]. There exist multiple signal transduction pathways in response to estrogen, and the same estrogen binding can lead to different, or even opposite [28,29], responses in ERα and ERβ (See Fuentes et al., 2019 [30] for a detailed review). Generally, ERα is known to modulate neurobiological reproductive systems, such as those involved in sexual characteristics and puberty. ERβ is known to be involved in the modulation of nonreproductive systems, such as anxiety, locomotion, fear, and memory and learning.

### 2.3. Estrogen Receptors in the Brain

Along with estrogens, ERα and ERβ are widely distributed in our brain, including the hippocampus, hypothalamus, amygdala, thalamic connectivity system regions [31] of the thalamus, cerebellum, and cortex, as well as the cortico-striato-thalamo-cortical (CSTC) circuit-related regions of the basal ganglia and striatum. GPERs are also expressed in the hippocampus, cortex, and hypothalamus [32]. Revealed by extensive neuroimaging studies, interestingly, these areas are the most frequently reported regions of deficits in psychiatric patients, and below, we provide a brief description of estrogen receptor distributions.

**Figure 1:**
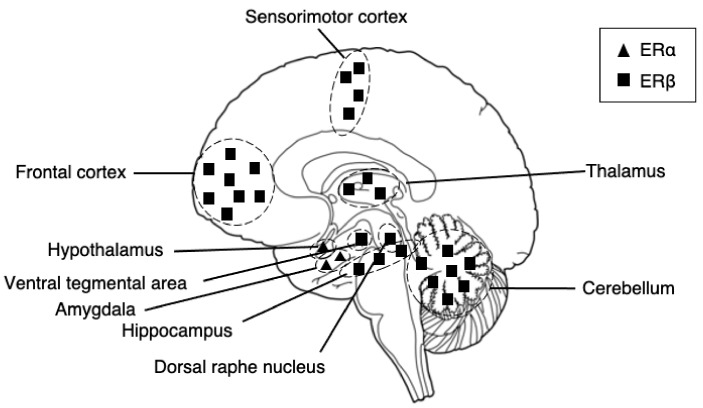
A schematic diagram of distributions of estrogen receptor alpha and estrogen receptor beta in our brains. The receptors have a different predominance of expression in distinct regions. ERα is predominantly expressed in the amygdala and hypothalamus, whereas ERβ is predominantly expressed in the somatosensory cortex, hippocampus, thalamus, and cerebellum.

The receptors possess different dominance in different brain regions (Figure 1). Describing the distribution in all brain regions may be unpractical for this article. However, to provide a brief description, in the cortex, ERα and ERβ are present particularly at the prefrontal and temporal cortexes in humans. In rats, reports show the existence of ERα in the medial prefrontal cortex [33] and the colocalization of ERα and ERβ in sensorimotor areas [34], with the density of ERβ being greater than that of ERα [35]. In the temporal cortex of humans, a report showed a higher density of ERα in the nuclei and ERβ in the cytoplasm [36]. In the hippocampus, the pivotal region of the cognitive functioning of learning and memory in our brain, ERβ is expressed at moderately high levels at the regions of the subiculum, cornu ammonis 1-2 (CA1-CA2), and CA3 dentate gyrus [35,37,38] and is a primary regulator of the region in both rats and humans. In the amygdala, ERα is the primary regulator of the region; thus, they are predominantly expressed [39]. In humans, ERα is found the highest at the periamygdala cortex, amygdala-hippocampal area, and posterior cortical nucleus [37,40]. The co-expression of both receptors is the highest at the medial posterior-dorsal nucleus [39]. ERα is also a primary regulator of the hypothalamus. In humans, the ERα is expressed the highest at the supraoptic, paraventricular, arcuate, and periventricular nuclei [37]. The regions both ERα and ERβ are co-expressed, although beta is expressed at low levels, at the supraoptic, paraventricular, arcuate, and ventromedial nuclei [37]. The expression of ERα and ERβ are found in the major node of the CSTC circuit in our brain, the basal ganglia, where also dopamine cell bodies reside. Reports show the expression of estrogen receptors in dopamine neurons in rodents [35,41] and even the modulation of dopamine neurotransmission by estrogen [42,43,44]. In rodents, reports have found ERα and ERβ expressions in the striatum, with estrogen receptors being expressed low at the nuclei and high at the extracellular sites [45]. However, ERα and ERβ expressions have not, so far, been detected in the human striatum [40]. Further evidence of ERα and ERβ exists in the center node of the thalamic connectivity system: the thalamus and the cerebellum. Both regions are primarily regulated by ERβ [46]. In the thalamus, in humans, low levels of ERβ are found in the paratenial and paraventricular nuclei, and ERα is found in the posterior nuclei only [47]. Interestingly, sex differences in these areas have been shown, with men expressing a higher density of nuclear ERβ receptors than women [47].

### 2.4. Mechanism of Actions on Neurotransmitter Systems

Mounting evidence from both clinical and preclinical studies suggests the modulation of estrogen, via estrogen receptor signaling, on neurotransmitter systems in our brains, such as dopaminergic, serotonergic, and glutamatergic, the key neurotransmitter systems implicated in major psychiatric disorders. Estrogens exert effects on neurotransmitter systems by targeting and regulating the expressions of specific subtypes of neurotransmitter system receptors in a region-specific manner, contributing to our cognition, mood, and behavioral responses. For example, estrogens present selectivity in exerting effects on serotonin receptor subtypes that have high implications to cognitive functions commonly disrupted across multiple psychiatric disorders, such as learning, memory, and cognitive flexibility [48,49]. Mounting reports suggest a strong modulatory effect of estrogen on major neurotransmitter systems in our brain, and extensive studies have found neuroleptic-like properties of estrogen [15] similar to atypical antipsychotics used in psychiatric disorders on dopaminergic, serotonergic, and glutamatergic systems. Thus, a better understanding of the nature of these interactions is suggested for assessing the therapeutic potential of estrogen. To maintain the scope of this article, brief descriptions will be provided in this article, but we recommend Krolick and her colleagues for a detailed review of the interactions [50].

Preclinical studies have extensively revealed many profound yet complex effects of estrogens on dopaminergic neurotransmissions [42]. Briefly, the current literature reports that (1) estrogens increase dopamine synthesis in the nucleus accumbens, induce presynaptic dopamine release in the striatum, and decrease the turnover in the nucleus accumbens [51,52,53,54,55,56]. (2) Evidence suggests the regulation of D1 and D2 receptor densities and functions by estrogens [57,58,59,60]. (3) Estrogens prolong neurotransmissions by reducing dopamine transporters in the nucleus accumbens [61,62,63]. Similarly, extensive studies report the effects of estrogens on the serotonergic neurotransmission system. Specifically, current evidence suggests that (1) estrogens upregulate the expression and activity of TPH to increase 5HT biosynthesis [51,64] and (2) regulate 5HT receptors 5HT2A and 2C, the receptors of which have high implications in depression [65,66,67,68,69,70]. (3) Estrogens regulate 5HT autoinhibition via the 5HT1A auto-receptor, resulting in an antidepressant-like activity [71]. (4) Estrogen treatments reduce the 5HT uptake to presynaptic cells and prolong neurotransmissions [72]. (5) Estrogens decrease 5HT metabolism via degradation by monoamine oxidase inhibitors (MAO) after 5HT is taken up into the presynaptic neurons [68,73,74]. It has also been shown that estrogens exert their effects on the glutamatergic neurotransmitter system, which facilitates most of our neurotransmissions in our brain and mediates our cognitive functions. Current reports suggest that estrogens affect N-Methyl-D-aspartic acid (NMDA) glutamate receptors and upregulate and increase their distributions [75,76,77]. Notably, reports revealed the neuroprotective effects of estrogen on cortical and hippocampal neurons against the effects of glutamate-mediated neurotoxicity [78,79].

### 2.5. Estrogen Receptors and Cognition

Extensive reports depict the effects of estrogens on cognition. In humans, it has been reported that verbal memory impairments and menopause-related cognitive decline can be rescued by estradiol replacement therapy [80,81]. Studies have been reporting varying the results of outcomes of estradiol replacement therapy, depending on the dosage, duration, and type of the treatment; however, in general, estrogens have beneficial impacts on cognitive functioning [81]. Finer details of the relationship have been thoroughly investigated in preclinical studies. Studies have reported the distinguished characteristics of estrogen receptors; ERβ knockout mice show severely disruptive behaviors in memory and learning [82], and ERα knockout mice show severe deficits in reproduction [83]. Further, their distributions and expressions in our brain regions converge onto most cognitively relevant brain regions, and, via estrogen signaling, they also exert effects on the synaptic formation [84]. For example, in a recent study, it was reported that ERβ plays a crucial role in motor learning in the cerebellum by potentiating the neuronal plasticity and synaptogenesis in that brain region [85].

Extensive reports suggest the particular involvement of estrogen on the working memory [86,87]. In an ovariectomy performed on rodents, both spatial and nonspatial working memory deficits were observed, and the estradiol treatment also rescued those deficits [88]. In humans, high estradiol levels during menstrual phases in healthy women and estrogen replacement therapy (ERT) in postmenopausal women have been shown to improve the spatial working memory [89,90]. Their notable actions on the hippocampus and prefrontal cortex, in particular, have also been reported. It has been reported, in rodents, that exogenous estradiol administration reverses the decreases in the dendritic spine density of neurons in the hippocampus and prefrontal cortex caused by an ovariectomy, as well as improving the memory [91,92,93]. The detailed actions are yet to be thoroughly elucidated; however, their receptor distributions are wide across brains, and complex interactions with multiple neurotransmitter systems, as described in previous sections, conveniently place them as a key player in cognitive functioning.

#### Beneficial Effects of Selective Estrogen Receptor Modulators (SERMs) on Cognition

Estrogen signaling and its effects on cognition are particularly relevant to psychiatric disorders, as they display global cognitive deficits; particularly the disorders possess different degrees of executive dysfunctions [94,95]. Nonetheless, despite vigorous effort, the currently available pharmaceutical treatments for psychiatric disorders—particularly, schizophrenia—do not show satisfactory results in treating the cognitive deficits. In schizophrenia, despite the cognitive deficits being related to the patient’s functional impairment, there exist mixed results in pharmaceutical treatments for cognitive deficits [96,97]. The alpha-7-nicotinic receptor agonist has shown significant beneficial effects with small effect sizes on the CogState battery [98,99]. However, both the alpha-7-nicotinic receptor agonist and modafinil have been found insignificant on the Measurement and Treatment Research to Improve Cognition in Schizophrenia [100,101]. Thus, currently, it is very urgent to identify novel pharmacological targets, and amongst many targets, estrogenic treatments have been showing highly promising results.

ERTs, which have beneficial effects on the domains of verbal memory, speech, abstract reasoning, and information processing in postmenopausal women, come with the side effects of increased risks of thromboembolism, hot flashes, and breast hyperplasia when used long term, and most importantly, the therapy is prohibited for use in men due to feminizing effects [102]. Thus, recent studies have been focusing on another class of drugs that act on the estrogen receptor, selective estrogen receptor modulators (SERMs), which have antagonistic effects in the breasts and uterus and agonistic effects in the bone and brain. There are two classes of SERMs: triphenylethylene, which includes tamoxifen, clomiphene, toremifene, and GW5407, and benzothiophene, which includes raloxifene, arzoxifene, bazedoxifene, and lasofoxifene. Each has different properties and treatment effects, depending on the estrogen receptor subtypes, coactivators, and corepressors in the brain region. Reports show SERMs interact with ERα, Erβ, and, also, GPER and can activate both genomic and nongenomic cascades, such as cAMP/PKA, MAPK/ERKs, PI3K/Akt, and Wnt/β-catenin, which are major signaling pathways in our brain for cognition and neuroprotection [103,104,105]. However, different SERMs, for their distinct properties, result in different actions in our brains. For example, tamoxifen, a first-generation SERM initially developed for the treatment of breast cancer, and raloxifene have shown a similar affinity for both ERα and ERβ, whereas raloxifene, a second-generation SERM developed for osteoporosis treatment, has a four-times higher affinity for ERα [106]. Thus, unlike their initial developmental purposes, studies found beneficial effects in cognition—particularly in the memory—as well as neuroprotective and antioxidizing effects in SERMs, both in healthy and injured brains [85,107,108,109,110]. Raloxifene, in particular, has been reported, via various cell signaling cascades, to regulate plasticity; improve memory; and exert neuroprotective, antioxidative, and anti-inflammatory effects [85,107,108]. Therefore, multiple clinical trials assessing their efficacies and effects on cognition have been conducted across multiple psychiatric disorders, described in the following sections.

### 2.6. Estrogen and Its Neuroprotective Effects

Converging lines of evidence report that estrogens, via estrogen signaling, are implicated in neuroprotection [111,112]. Evidence suggests their implications in synaptic plasticity, antioxidative effects, apoptosis, and protection against excitotoxicity [113,114,115,116]. Reports also have shown estrogens facilitate glucose metabolism by having a regulatory role in the cerebral blood flow and can enhance the electron transport chain activity to provide more energy to neurons [117].

Further, estrogens provide neuroprotection by having anti-inflammatory effects [118,119]. They regulate and promote the synthesis of neurotrophins, such as brain-derived neurotrophic factor (BDNF), which is a highly implicated molecule to various psychiatric disorders for its pertinent roles in neuronal survival, differentiation, and synaptic plasticity [120]. Further, reports have shown that ERα and ERβ have regulatory roles in the production of proinflammatory cytokines and chemokines and that this can occur either through estrogen-dependent or -independent mechanisms [121]. However, there exist “critical periods” for estrogens to exert their neuroprotective effects. It has been reported that estrogen therapies need to be given immediately after brain injuries, as the treatment loses the effect when given ten weeks post-ovariectomy [122]. In the same study, long-term estrogen deprivation caused a reduction in ERα receptors in the hippocampus, and the “critical period” is suggested to be due to tissue-specific reductions of estrogen receptors [122].

## 3. Estrogen Receptor and Psychiatric Disorders

### 3.1. Schizophrenia

Schizophrenia is a severely debilitating disorder that affects 1% of the population. It has, largely, three symptom domains of positive symptoms, negative symptoms, and cognitive deficits. Sex differences in the pathophysiology are well-documented in the literature. Compared to women, in males, evidence shows higher incidence rates, early-onset, and different symptoms [123]. Men have earlier onset and higher incidence rates of the disorder than women, as well as present more symptoms of conduct disorders, aggression, antisocial personality traits, and higher levels of psychopathology. Women have a higher incidence of negative symptoms, substance abuse, and depression. Further, there exist differences in the number of peaks in the age of onset between the sexes. Men have a single peak between 21 and 25 years of age, and women have two, the first after menarche and the second postmenopause [124]. This has led to the “estrogen hypothesis” in schizophrenia, which posits that estrogens provide neuroprotective effects against the disorder in regards to the onset, progression, and symptom severity, as well as the promotion of healthy brain development [125,126,127].

Studies revealed and confirmed detailed correlations between estrogen levels and schizophrenia symptoms. Low plasma estrogen levels have been correlated with increased risks for schizophrenia symptoms in women [128], and estrogen levels across the menstrual cycle have been inversely correlated with psychopathological symptoms in women with schizophrenia [129]. Menstrual cycle irregularities in schizophrenia patients have also been reported to be a predictor of lower cognitive performance in areas of psychomotor speed, verbal fluency, and verbal memory, suggesting that cognitive deficits in schizophrenia are partly attributed to estrogens [130]. During pregnancy, when in the surge of estrogen levels, patients have shown low rates of relapse of the disorder [131]. Studies also have found that the early timing of menarche has beneficial effects in providing neuroprotective effects against psychosis deterioration [132], and a recent neuroimaging study revealed that an earlier age at menarche (i.e., earlier availability of estrogens) results in more normative hippocampal connectivity in high risk for psychosis youths [133]. A study also suggested the neuroprotective role of estrogen in reducing symptom severity and susceptibility [134].

Studies have also revealed the effects of estrogens on cognitive deficits seen in schizophrenia patients. Both in healthy populations and in schizophrenia patients, studies have shown that estrogen levels correlate with well-being and cognitive functioning. Further, reports have demonstrated low estradiol phases are associated with poorer verbal and spatial memory, as well as perceptual-motor speed [135]. A neuroimaging study using functional magnetic resonance imaging (fMRI) also reported that there is a significant positive correlation between sex steroid levels and brain activity in both female schizophrenia patients and healthy males [136]. In preclinical studies, using rodent models of schizophrenia, a few studies have examined the molecular and genetic details of such cognitive disruptions seen in patients. The studies yielded promising results that estrogens can be used to ameliorate working memory deficits. Different models exist, but most tried to implement the cognitive deficits observed in schizophrenia by manipulating the NMDA receptors. Celia Moreira Borella and colleagues [137] reported working memory and prepulse inhibition (PPI) deficits when estrogen levels are the lowest and normal behaviors when the levels are the highest in a model using a neonatal *N*-Methyl-d-aspartic acid receptor (NMDAR) blockade with ketamine. Gogos and colleagues [138,139] reported the effects of estrogen or selective estrogen receptor modulators on PPI deficits caused by MK801 or apomorphine in ovariectomized rats. Further, in a recent study, Gogos and colleagues [140] reported that chronic treatment with estrogens reversed the PPI disruptions and the increased dopamine D2 receptor-binding densities in Poly(I:C)-treated rodents, suggesting that the beneficial effects may be mediated by selective changes in densities of dopamine D2 receptors.

Mounting evidence exists reporting subnormal estrogen levels in both treated and untreated schizophrenia patients and high risk for psychosis subjects [136,141,142]. In addition to the aforementioned deficits observed peripherally, studies exist reporting alterations in the brain’s response to these hormones. It has been reported that both men and women with schizophrenia have reduced mRNA levels of ERα in the hippocampus [143]. The ERα gene and its mRNA expression has further been reported to be associated with schizophrenia [144]. Further, reports have also shown that women with schizophrenia often are hypoestrogenic, and converging evidence suggests this may be the consequential effect of hyperprolactinemia, a common side effect of antipsychotic mediation [145]. It has been suggested that, as increased levels of prolactin suppress the hypothalamic-pituitary-gonadal (HPG) axis in a negative feedback manner, estrogen and testosterone levels become decreased, resulting in hypoestrogenism observed in schizophrenia patients. However, recent lines of investigation revealed that hyperprolactinemia alone cannot be a full explaining factor of hypoestrogenism in schizophrenia [146] and that hyperprolactinemia is also independent of antipsychotics [147,148,149,150] (see Du and Hill, 2019 for a detailed review [151]).

Thus, currently, clinical trials are being conducted to test estrogen as a new target of therapy. Initially, studies focused on rescuing the estrogen level itself. The direct administration of estrogens showed improvements in the speech comprehension of female schizophrenia patients [152]. Transdermal estradiol patch therapy also demonstrated beneficial effects and significantly improved the psychotic symptoms in female patients with schizophrenia; however, no positive effects were found in their cognitive functioning [153]. Recent research paradigms have shifted towards assessing SERMs—in particular, raloxifene—on symptom amelioration and cognition enhancements in schizophrenia patients for their lack of sensitization and feminization and selective action as potent estrogens only in the bone and brain.

Raloxifene has shown promising results in the improvement of the cognitive impairment—particularly, attention, memory, and learning—seen in schizophrenia. Further, raloxifene improves symptoms in schizophrenia patients. Recent studies have found that raloxifene improves both positive and negative symptoms in women and negative symptoms in men [11]. Its effects were also assessed in multiple clinical trials in which its beneficial effects on multiple domains of executive functions and psychopathology were confirmed in various subgroups of schizophrenia patients, such as men and women with schizophrenia, treatment-resistant young women with schizophrenia, and postmenopausal women with schizophrenia [154,155,156,157]. Further, in one study, the beneficial effects were maintained even when the dose was reduced to half [156,158]. However, more studies are needed to elucidate the mechanisms of raloxifene, and the current literature shows varying degrees of improvements by raloxifene on cognition and psychopathology, suggesting further clinical trials.

### 3.2. Bipolar Disorder

Bipolar disorder is characterized by cycles of mania and depression. The disorder can be classified into Bipolar I disorder, which is characterized by much severer mood episodes, from mania to depression, and Bipolar II disorder, which is characterized by milder episodes of hypomania and alternate with severe depression. There exist gender differences in the disorder in that women present with symptoms later in life than men and have faster cycling of mania and depression than men (Table 1). Gender differences are also seen amongst the subtypes. Bipolar II disorder is more common in women than men. Numerous reports show that women with bipolar disorder, during periods of hormonal fluctuation, are associated with increased vulnerability to developing depression and increased risk of affective dysregulation.

Increased levels of GPER-1 were recently reported in euthymic outpatients of bipolar disorder, the results of which were not influenced by medications [159]. So far, the two studies that have examined the relationship between ERα, ERβ, and bipolar disorder have found negative results [160,161], unlike in schizophrenia. Despite there being a limited number of studies examining the associations between estrogens and estrogen receptors in bipolar disorder, reports show high associations between the symptomatic course in patients with bipolar disorder and the periods of hormonal fluctuations. It has been reported that bipolar disorder patients who experience premenstrual exacerbation are more likely to have a worse course of illness, a shorter time to relapse, and increased severity in their symptoms [162]. Further, in a recent study, the bipolar patients who report reproductive cycle event-related worsening of their mood were associated with rapid cycling, comorbid anxiety, and mixed mood episodes [163]. Taken together, the current lines of evidence show that disruptions in estrogen and estrogen signaling and estrogen fluctuations are associated with the symptoms.

Several clinical trials on SERMs have been showing promising effects in the treatment of bipolar disorder. Tamoxifen has been shown to have effects in reducing mania and depression when used together with a lithium treatment in children and adolescents with acute mania [164]. The study also reported the high efficacy of tamoxifen despite the small sample size. In a meta-study, tamoxifen adjuvant therapy was reported to reduce the frequency of manic episodes in bipolar patients [165]. However, tamoxifen is known to have side effects of thromboembolic events and increasing risks of endometrial cancer. Thus, efforts have been put into understanding and elucidating the detailed mechanisms of the actions of the drug. Animal studies have reported that the beneficial effects of tamoxifen on mania from the coadministration of lithium and tamoxifen come partly from lithium and tamoxifen changing the protein kinase C signaling pathway [166]. The current literature, however, lacks the long-term effects of tamoxifen, and further studies are warranted.

### 3.3. MDD

MDD can be chronic or recurrent, and its impacts on mood and behavior are associated with poor health and mortality. Gender differences exist in MDD, like other psychiatric disorders, and women have a higher prevalence than men.

It is speculated, with multiple lines of evidence, that alterations in hormones play a crucial role in the pathophysiology of the disorder. Reports show high associations between the symptomatic course in patients with MDD and the periods of hormonal fluctuations. The patients, when in periods of ovarian hormone withdrawal, such as a postpartum period or menopause, have increased risks of mood symptoms and the occurrence of MDD [167]. At a molecular level, the GPER level has been reported to be elevated in MDD compared to healthy subjects, which also correlated with depression scores [168]. Using the data from one million Danish women, oral contraceptive uses were associated with an increased risk of a depression diagnosis, antidepressant treatments, and suicidal acts [169,170]. Further elucidation into detailed mechanisms involved in such a phenomenon was made in animal studies. During low-estradiol times of the cycles, rodents showed more profound depressive-like behaviors [171]. Further, ovariectomy caused an enhanced feeling of despair and was rescued with the estradiol administration in rodents [172].

ERTs are currently used to treat peri- or post-MDD patients. However, currently available clinical trials examining moods in pre- and postmenopausal women treated with hormone replacement have yielded mixed results for wide variations in the symptomatology of recruited samples and treatment timing postmenopause across studies [17]. It was revealed in a study that the early treatment of ERT has a cardioprotective effect, whereas the same treatment when treated 10 years after menopause exerted risk-enhancing effects [173].

### 3.4. ASD

ASD is a neurodevelopmental disorder that begins early in childhood. The disorder is characterized by dysfunctions in communicating and interacting with others, as well as learning disabilities. With the prevalence ratio of 4:1, men are more highly likely to develop this disorder [174]. Reports have long been made for the association between ASD development and increased testosterone exposure during pregnancy [175]. Studies associate testosterone levels to various symptoms and cognitive deficits manifested in ASD, such as social anxiety and reduced empathy, as well as deficits in social and language developments in ASD patients [68,176]. These led to the “extreme male brain” (EBM) theory [177], which proposes that ASD patients, due to elevated prenatal testosterone levels, can be considered as having an extreme of the normal male profile for their cognition and show a strong predominance of systemizing over empathizing.

The literature also describes estrogen-signaling disruptions in ASD. Aromatase, CYP19A1, which converts testosterone to estradiol, as well as estrogen and estrogen receptors, are reported to be decreased in ASD patients [178,179,180]. There exist significant associations between the ERβ gene and autism trait, measured by the Autism Spectrum Quotient and the Empathy Quotient in ASD patients [180]. Further, in a recent study, ERβ mRNA and protein levels were reported to be reduced at the middle frontal gyrus in the postmortem brains of ASD subjects. In the same study, ER coactivators were also reported to be disrupted in ASD. There were impairments in the steroid receptor coactivator-1, CREB-Binding Protein (CBP), and P/mRNA levels in ASD patients [179]. With recent studies reporting disruptions in estrogen and estrogen signaling in ASD, thus, it may potentially be that abnormal levels of testosterone and the testosterone-associated cognitive deficits and symptoms may be representing one of the factors of ASD risk. Put together, the consideration of both testosterone and estrogen, for their close relationship, may benefit identifying the risk factors of ASD.

### 3.5. ADHD

ADHD is a neurodevelopmental disorder characterized by marked deficits in attention, hyperactivity, and impulsivity. Gender disparities in this disorder include males having twice as likely prevalence than females and ADHD females having increased inattentive symptoms than males [181], although the underlying factors are not well-elucidated.

Despite the associations between ADHD and estrogen signaling remaining relatively unexplored yet, a few case studies support the association. ADHD symptoms exacerbate a week before menstruation and become alleviated during pregnancy [182]. A recent study investigating serum estrogen and GPER levels in children with ADHD reported comparable serum estrogen levels but reduced GPER in ADHD children [183]. Much literature lies in the investigation of bisphenol A (BPA). BPA is a xenoestrogen compound that binds to estrogen receptors and affects the downstream cell signaling cascade [184]. Evidence supports associations between BPA and ADHD-like symptoms, occurring via disrupting multiple neurotransmitter systems of catecholaminergic, dopaminergic, and serotonergic signaling systems [185,186]. It has shown effects in behavioral outcomes in ADHD children when used in high doses [187]. Further, a study reported a significant positive association between BPA exposure and ADHD risk at four years of age, although the effect disappeared by seven years of age [187]. A recent meta-analysis examined the prenatal exposure to BPA in ADHD children and rodent models. Early BPA exposure was associated with increased hyperactivity in male rodents and both males and females in humans [188]. Given the contribution of estrogen to executive function, a possible marker of ADHD, the current literature warrants further studies exploring the contributions of estrogen in ADHD pathophysiology.

### 3.6. Anxiety Disorders

#### 3.6.1. Generalized Anxiety Disorder (GAD)

GAD is characterized by excessive, ongoing anxiety and worry that interferes with daily functioning. The disorder is twice more prevalent amongst women than men [189], and interestingly, it is developed and manifested after puberty [190], suggesting contributing roles of hormones—particularly, estrogen—to the disorder pathophysiology.

Indeed, studies report disruptions of estrogen signaling in GAD. A study reported increased GPER levels in GAD, which further correlated with the anxiety severity in patients irrespective of gender [191]. In rodents, GPER-deficient rats showed anxiety-like behaviors, as well as low corticosterone [192]. Interestingly, reports show ERβ signaling has anxiolytic effects [193], and in mice, ERβ deficiency has been associated with social and mood-related behavioral disturbances via the oxytocin and arginine-vasopressin signaling pathways [194]. A recent report investigated interactions with the glutamatergic system and estrogen and showed that, for the estrogen mitigation of anxiety-related behaviors in rats, mGlu5 activation is necessary [195].

#### 3.6.2. PTSD

PTSD is developed after experiencing a traumatic event and is characterized by severe anxiety, flashbacks, and nightmares of the trauma. Women have a twice-higher prevalence of the disorder than men following trauma [196]. Further, a meta-analytic study of 48 studies reported women to have better treatment responses than men [197]. Although the characteristics of the traumatic events may be different amongst the genders (e.g., women experience a greater number of interpersonal and sexual violence events, while men experience a greater number of industrial accidents and war), these are not completely explanative of the disparities. Recent studies suggest a high involvement of sex hormones—particularly, estrogen—in the pathophysiology and treatment of PTSD [198,199].

Reports have shown that PSTD symptoms fluctuate with estrogen levels. One study reported increased phobic anxiety and depression at cycles of low estrogen levels [200]. Multiple genetic studies further support the implications of estrogen signaling in PTSD. The pituitary adenylate cyclase-activating peptide receptor gene has been reported to be associated with PSTD symptom severity in women but not men [201]. DNA methylation of the histone deacetylase 4 (HDAC4) gene, which is estrogen-dependent, is associated with fear learning and memory in PTSD [202]. It has also identified the implications of the ERα genes rs2234639 and rs9340799 in PTSD [203,204]. Recently, a neuroimaging study, using functional magnetic resonance imaging, administered blocks of the fear condition and extinction training to PTSD patients and measured their responses to fear with a skin conductance response. The study revealed the modulatory role of estrogens in PTSD severity and the arousal response, such that higher estrogens have protective effects against the negative impacts of PTSD symptoms [205]. This evidence, taken together, suggests a promising outlook towards using estrogen or the estrogen-signaling pathway as a putative pharmacological adjunctive treatment [199].

### 3.7. Eating Disorders

Eating disorders comprise the development of unhealthy eating habits due to psychological conditions. There exist gender disparities in the disorders, in that females have 3-10 times higher prevalence than men [206]. Sociocultural factors, indeed, are a significant factor driving the huge disparity; however, animal studies have shown pronounced differences in the disorder phenotypes occurring during puberty, supporting a big part of the biological factors—particularly, hormones—contributing to the disorder pathophysiology [207,208].

Preclinical studies have revealed that perinatal exposure to testosterone causes the sexual differences in behaviors of food intake, as well as the preference for sweet tastes [209]. In a study where the genetic influences on binge eating in girls were examined, it was found that girls with relatively high estrogen levels had minimal genetic influences of binge eating. On the other hand, girls with relatively low estrogen levels have greater genetic influences on binge eating [210], suggesting a protective role of estrogen against the genetically-mediated eating disorder. Further, genetics studies have identified risk the genes for an eating disorder. The ERα gene has been identified as being associated with an eating disorder, and a study reported that the decreased gene activity increased the risk of developing an eating disorder [211]. Further, the HDAC4 gene, of which DNA methylation is dependent on estrogen, was found also associated with eating disorders by changing feeding behaviors in mice [211]. Nonetheless, the pharmacological treatments for eating disorders, including estrogen or estrogen signaling target treatments, have, so far, been underexplored [212].

### 3.8. Substance Use Disorder

Substance use disorders are characterized by the inability to control using legal or illegal drugs or medication, such as marijuana, stimulants, and heroin, as well as nicotine, and alcohol. Men have a higher tendency to use illicit drugs and alcohol than women. Further differences lie in the treatment adherence, illness course, and comorbidities. For example, women generally seek help earlier and have a higher prevalence of comorbid psychiatric disorders than men [213].

#### Alcohol Use Disorder

Amongst the different substances in substance use disorders, most literature exists in investigating alcohol use disorder. Men and women have different reasons for binge drinking. Women drink for self-medication and soothing mood disturbances [213]. Further, women often have worse health outcomes from the abuse, including liver disease and brain damage [214,215].

Studies have reported estrogen and estrogen signaling involved in alcohol-abusing behaviors. The estrogen level is positively associated with alcohol consumption [216] in humans and in rodents [217]. Further, a recent study showed that ERα promotes the ethanol response of ventral tegmental area neurons, the process of which requires mGLuR1 activity. To add more, the study observed a more dramatic effect of ERα reduction in the ventral tegmental area (VTA) on binge-like drinking behavior than ERβ. However, the effect was only observed in female mice and not male mice, providing evidence that alcohol use disorder treatments may need to take into account genders [218].

## 4. Conclusions

Extensive literature supports estrogen and estrogen-signaling disruptions across the psychiatric illnesses of schizophrenia, bipolar disorder, MDD, ASD, ADHD, GAD, PTSD, eating disorders, and substance use disorders. Estrogens and estrogen signaling play a pertinent role in the regulation of neurotransmitter systems, such as dopaminergic, serotonergic, and glutamatergic, and actively participate in cognitive functioning—most importantly, memory. Further, they provide neuroprotective and anti-inflammatory effects. Estrogen and estrogen signaling are disrupted in multiple psychiatric disorders, with varying degrees of disruptions affecting different downstream cell cascades. Future studies elucidating estrogen and estrogen-signaling disruptions and possible novel treatment strategies in major psychiatric disorders are warranted.

## Figures and Tables

**Table 1:** Gender and hormonal effects across psychiatric illnesses.

	Prevalence(Male:Female)	Menstrual Cycle Effects	Effects of Menopause
Schizophrenia	1.4:1	↑ sx in low hormone phase	↑ sx , ↑ prevalence
Bipolar disorder	1:1	↑ sx in low hormone phase	↑ sx
MDD	1:1.6	↑ sx in low hormone phase	↑ sx , ↑ prevalence
ASD	1:4	↑ sx in low hormone phase	↑ sx
ADHD	1:2	↑ sx in low hormone phase	↑ sx , ↑ prevalence
GAD	2:1	↑ sx in low hormone phase	↑ sx , ↑ prevalence
PTSD	2:1	↑ sx in low hormone phase	↑ prevalence
Eating disorder	3 to 10 times in women	↑ sx in low hormone phase in bulimia,N/A in anorexia	↑ sx , ↑ prevalence
Substance use disorder	1:2	Reinforcing effects of stimulants (Estrogen, ↑;Progesterone, ↓)	↑ prevalence in alcohol abuse

Major depressive disorder (MDD); Autism spectrum disorder (ASD); Attention-deficit/hyeractivity disorder (ADHD); General anxiety disorder (GAD); Post traumatic stress disorder (PTSD); Symptom (Sx); Not available (N/A); Increase (↑); Decrease (↓).

## Abbreviations

ADHD	Attention-deficit hyperactivity disorder
Akt	Protein kinase B
ASD	Autism spectrum disorder
BDNF	Brain-derived neurotrophic factor
BPA	Bisphenol A
CA1	Cornu ammonis 1
CA2	Cornu ammonis 2
CA3	Cornu ammonis 3
cAMP	Cyclic adenosine monophosphate
CSTC	Cortico-striato-thalamo-cortical
ERα	Estrogen receptor alpha
ERβ	Estrogen receptor beta
ERE	Estrogen response elements
ERK	Extracellular signal-regulated kinase
ERT	Estrogen replacement therapy
fMRI	Functional magnetic resonance imaging
GPER	G-protein coupled receptor 30
HDAC4	Histone deacetylase 4
MAPK	Mitogen-activated protein kinase
NTD	Amino-terminal domain
PI3K	Phosphatidylinositol 3-kinase
PKA	Protein kinase A
SERMs	Selective estrogen receptor modulators
Wnt	Wingless-int
